# Exosomes as potential alternatives to stem cell therapy for intervertebral disc degeneration: in-vitro study on exosomes in interaction of nucleus pulposus cells and bone marrow mesenchymal stem cells

**DOI:** 10.1186/s13287-017-0563-9

**Published:** 2017-05-10

**Authors:** Kang Lu, Hai-yin Li, Kuang Yang, Jun-long Wu, Xiao-wei Cai, Yue Zhou, Chang-qing Li

**Affiliations:** 1 Department of Orthopedics, XinQiao Hospital, Third Military Medical University, Chongqing, China; 2 Department of Dermatology, XinQiao Hospital, Third Military Medical University, Chongqing, China

**Keywords:** Intervertebral disc degeneration, Exosomes, Mesenchymal stem cell, Nucleus pulposus cell, Differentiation, Proliferation, Migration

## Abstract

**Background:**

The stem cell-based therapies for intervertebral disc degeneration have been widely studied. However, the mechanisms of mesenchymal stem cells interacting with intervertebral disc cells, such as nucleus pulposus cells (NPCs), remain unknown. Exosomes as a vital paracrine mechanism in cell–cell communication have been highly focused on. The purpose of this study was to detect the role of exosomes derived from bone marrow mesenchymal stem cells (BM-MSCs) and NPCs in their interaction with corresponding cells.

**Methods:**

The exosomes secreted by BM-MSCs and NPCs were purified by differential centrifugation and identified by transmission electron microscope and immunoblot analysis of exosomal marker proteins. Fluorescence confocal microscopy was used to examine the uptake of exosomes by recipient cells. The effects of NPC exosomes on the migration and differentiation of BM-MSCs were determined by transwell migration assays and quantitative RT-PCR analysis of NPC phenotypic genes. Western blot analysis was performed to examine proteins such as aggrecan, sox-9, collagen II and hif-1α in the induced BM-MSCs. Proliferation and the gene expression profile of NPCs induced by BM-MSC exosomes were measured by Cell Counting Kit-8 and qRT-PCR analysis, respectively.

**Results:**

Both the NPCs and BM-MSCs secreted exosomes, and these exosomes underwent uptake by the corresponding cells. NPC-derived exosomes promoted BM-MSC migration and induced BM-MSC differentiation to a nucleus pulposus-like phenotype. BM-MSC-derived exosomes promoted NPC proliferation and healthier extracellular matrix production in the degenerate NPCs.

**Conclusion:**

Our study indicates that the exosomes act as an important vehicle in information exchange between BM-MSCs and NPCs. Given a variety of functions and multiple advantages, exosomes alone or loaded with specific genes and drugs would be an appropriate option in a cell-free therapy strategy for intervertebral disc degeneration.

## Background

According to the Global Burden of Disease (GBD) report, lower back pain (LBP) has been the leading cause of years lived with disability (YLDs), which contributed 10.7% of total YLDs and rose by 42.6% in the last 20 years (1990–2010) [1]. Although there are numerous potential pain generators in the lumbar spine, symptomatic disc degeneration is considered a significant contributor to LBP. At present, the primary therapeutic methods for degenerative disc diseases (DDD) are conservative treatment, surgical discectomy, intervertebral disc displacement and, as a last resort, spinal fusion. Despite discectomy providing a favorable outcome in most patients, recurrent disc herniation might be an inevitable issue arising for both surgeons and patients. Meanwhile, it is common sense that the spinal fusion could result in new clinical entities, such as adjacent segment diseases and revision spinal fusion. Therefore, in the past decades, many studies have been carried out to find a solution in the field of molecular biology and stem cell therapy.

As a worldwide issue, therapeutic methods for IVD degeneration have achieved great progress. While a number of approaches have been considered, MSC-based regenerative therapies offer huge potential. The rationales of stem cell therapy are known as replenishing intervertebral disc cells via multipotent differentiation, promoting nucleus pulposus cell (NPC) proliferation, reducing apoptosis, resisting inflammation and reinforcing immune privilege [2]. In an in-vitro study, MSCs were cocultured with NPCs by direct cell–cell contact or noncontact methods [3, 4]. They differentiated along the NP lineage and simultaneously stimulated the degenerate NPC population to regain a normal (nondegenerate) phenotype [4, 5]. However, the mechanisms by which NPCs and MSCs interact in this system are currently unclear.

Cell–cell communication is based on paracrine, connexons or cell–cell direct contact. In recent years, researchers turned attention to a special paracrine mechanism: that of exosomes. Exosomes have been found in almost all biological fluids, such as blood, sperm, milk and urine, and are defined as “extracellular vesicles released on exocytosis of multivesicular bodies (MVBs) filled with intraluminal vesicles (ILVs)” [6–8]. Exosomes are nanosized membrane vesicles with a diameter of 30–100 nm [9, 10] (or 40–120 nm [8]) which originate from multivesicular endosomes and are released by all kinds of cells into the extracellular environment. It has been reported that there are multiple contents in exosomes including cytokines, proteins, lipids, mRNAs, miRNAs, noncoding RNAs (ncRNA) and ribosomal RNAs [8]. As a consequence of their endosomal origin, nearly all exosomes contain proteins involved in membrane transport and fusion (e.g., Rab GTPases, Annexins, flotillin), MVB biogenesis (e.g., Alix and TSG101) and the processes requiring heat shock proteins (HSP70 and HSP90), integrins and tetraspanins (e.g., CD63, CD9, CD81 and CD82) [8, 11, 12]. The bilayer membranous shell of exosomes prevents degradation of their contents [6], so the contents of exosomes are more stable during long-distance transportation and interaction with target cells. It is interesting that exosomes play unique roles in cell communication based on their cell origin; for example, MSC-derived exosomes may provide cell-free therapy as an alternative to the traditional MSC therapy [13]. In this study, we investigated the role of exosomes in the interaction of NPCs and BM-MSCs.

## Methods

### Cell culture

Human NPCs were isolated and grown as described previously [14]. Briefly, NP tissues were obtained from 10 patients who underwent transforaminal endoscopic surgery for lumbar degenerative disease. Annular fibers (AF) and cartilaginous endplate (CEP) in the specimens were removed under microscopy. The NP tissues were washed with PBS repeatedly and then digested with 0.5% type II collagenase (Sigma, St Louis, MO, USA) for 6 hours. Tissue debris was then removed by passing through a 75-μm filter. The resulting cells were centrifuged at 250 × *g* for 10 min and resuspended in Dulbecco’s modified Eagle medium/F12 (DMEM/F-12) medium (Gibco, Grand Island, NY, USA) with 10% fetal bovine serum (Gibco) and 100 U/ml penicillin–streptomycin. Finally, the NPCs were incubated at 37 °C in a humidified atmosphere of 5% CO_2_. Human bone marrow-derived mesenchymal stem cells (BM-MSCs) (Cyagen Biosciences Inc.) were cultured in Growth Medium for Human MSCs (OriCell™; Cyagen Biosciences Inc.) at 37 °C in 5% CO_2_. The study was approved by the Medical Ethics Committee of the Second Affiliated Hospital of Third Military Medical University, PLA. Written informed consent was obtained from all patients.

### Isolation and purification of exosomes

Exosome purification involves several centrifugation and ultracentrifugation (Himac cp80wx/P70A-980) steps as described previously [6, 15–17]. Exosome-depleted medium was prepared by overnight ultracentrifugation of conditioned medium at 100,000 × *g*, 4 °C. BM-MSCs and NPCs were respectively seeded at 1 × 10^6^ cells per 25-cm^2^ dish and cultured in 5 ml of exosome-free medium. The medium was collected after 48 hours. For isolation and purification of exosomes, the medium was centrifuged at 300 × *g* for 10 min and 2000 × *g* for 10 min to remove cells and dead cells. The supernatant was then filtered through 0.22-μm membrane filters with pressure to remove cell debris and vesicles larger than 220 nm in diameter. Next, exosomes were pelleted by ultracentrifugation at 100,000 × *g* for 70 min. Finally, the pellet was washed and resuspended in PBS [16–19]. The exosomes were quantified by BCA protein assay (Beyotime Biotechnology) and cryopreserved at –80 °C.

### Transmission electron microscopy

Exosomes obtained after differential centrifugation of conditioned cell-culture medium were suspended in PBS. Ten micrograms of exosome suspension was loaded onto formvar carbon-coated 200 mesh copper grids for 10 min at room temperature. Excessive fluid was slightly drained with filter paper. Adsorbed exosomes were negatively stained with 1% phosphotungstic acid for 5 min. Finally, the air-dried exosome-containing grids were observed by transmission electron microscope (JEM-1400PLUS, Japan) operating at 100 kV.

### SDS-PAGE and western blot analysis

BM-MSCs, NPCs and exosomes derived from the two kinds of cells were lysed using RIPA (Beyotime Biotechnology) containing 50 mM Tris (pH 7.4), 1% Triton X-100, 150 mM NaCl, 1% sodium deoxycholate, 0.1% SDS, EDTA, sodium orthovanadate, sodium fluoride, leupeptin and 1 mM PMSF. Cellular lysates and exosomal lysates were subjected to SDS-PAGE and transferred to a PVDF membrane (0.45 μm; Millipore). After blocking in 5% nonfat milk in TBST, PVDF membranes were incubated with anti-CD63, anti-Tsg101 and anti-Calnexin [7] (Abcam) solution (1:1000 dilution) overnight at 4 °C, respectively. Membranes were washed in TBST three times, and incubated with a horseradish peroxidase-conjugated secondary antibody (1:1000 dilution) for 2 hours. All membranes were visualized using chemiluminescence substrate (Pierce Biotechnology).

### Labeling exosomes with PKH67

Exosomes were labeled with PKH67 (Sigma-Aldrich) according to the manufacturer’s protocol. Briefly, 250 μg of exosomes was resuspended in 1 ml of diluent C (Sigma-Aldrich) and mixed with PKH67 diluted in Diluent C for a final concentration of 2 × 10^–6^ M PKH67. The exosomes was incubated in dye suspension for 5 min. Excessive dye from the labeled exosomes was neutralized with 2 ml of 5% BSA/PBS. Finally, the supernatant was removed by centrifugation (100,000 *g* for 70 min at 4 °C) and resuspended in 50 μl PBS. A mixture without exosomes was used as a negative control to examine any carryover of PKH67 dye. For the negative control, labeling was performed as described but without exosomes.

### Fluorescence confocal microscopy

PKH67-labeled exosomes derived from BM-MSCs were coincubated with NPCs for 4 hours. Equally, PKH67-labeled NPC exosomes were coincubated with BM-MSCs for 4 hours. Cellular nuclei were stained by DAPI. Imaging of exosomes uptake was performed by a fluorescence confocal microscope. Images were analyzed with Leica Application Suite Advanced Fluorescence (LAS AF) software.

### Cell proliferation test (Cell Counting Kit-8)

To value the capacity of BM-MSC exosomes to facilitate NPC proliferation, the Cell Counting Kit-8 assay was used. NPCs were seeded on 96-well cell culture cluster plates (Corning Inc., Corning, NY, USA) at a concentration of 2 × 10^3^ cells/well in volumes of 100 μl and grown overnight. The duration of BM-MSC exosome interaction with NPCs was grouped into 3, 6, 9 and 12 days and a control group (NPCs alone without BM-MSC exosomes). To eliminate the detection error, all groups (including the control group) were cultured for 12 days under the same condition and the BM-MSC exosomes (50 μg/ml, resuspended in 100 μl DMEM/F-12 medium) were added to the NPCs at the due time according the timing of the experimental design. Finally, 10 μl of Cell Counting Kit-8 reagents (Dojindo, Kumamoto, Japan) was added to each well at the due time points and then incubated for another 2 hours at 37 °C in the dark. The optical density (OD) value was measured at 450 nm, and three trials were performed to obtain the average values. The relative proliferation rate to control was used to create the cell growth chart and examined using one-way ANOVA.

### In-vitro BM-MSC migration assay

The chemotactic response of BM-MSCs to NPC exosomes was investigated using a transwell migration assay (8-μm pore membrane; Millipore). BM-MSCs were starved in serum-free Growth Medium for Human MSCs (OriCell™; Cyagen) for 24 hours and then transferred to the upper chambers (2 × 10^5^ cells) in 100 μl of serum-free Growth Medium for Human MSCs (OriCell™; Cyagen). Then 10 μg/ml NPC exosomes in 500 μl of Growth Medium for Human MSCs were added to the lower chamber, while the Growth Medium for Human MSCs without NPC exosomes was added to the control group. After 10-hour incubation at 37 °C and 5% CO_2_, the membranes were fixed in 4% paraformaldehyde and stained with 0.1% crystal violet. The numbers of migrated cells in nine random fields were counted using a microscope (×200).

### Quantitative RT-PCR analysis of gene expression

We used quantitative RT-PCR analysis to evaluate the expression of NPC phenotypic genes (*ACAN*, *SOX-9*, *COL2A1*, *CA12*, *HIF-1α* and *KRT19*) in BM-MSCs which have been stimulated by NPC exosomes. After 3, 7, 10 and 14 days of stimulation, BM-MSCs were rinsed in PBS and lysed in TRIzol (Invitrogen). Total RNA was then extracted following the manufacturer’s instructions. The concentration of total RNA was determined with a spectrophotometer at 260 nm wavelength. Two micrograms of total RNA was used to synthesize cDNA. The primers for detecting differentiation-related genes are presented in Table 1. β-actin was used to normalize the level of the interest genes. For qRT-PCR analysis, the reaction system (20 μl) including 10 μl of SYBR Green (BIO-RAD), 2 μl of cDNA, 1 μl of each primer and 6 μl of sterile distilled water was performed in triplicate with the CFX96 Touch™ Real-Time PCR Detection System (BIO-RAD) for PCR amplification. Target gene expression was calculated by the 2^–ΔΔCt^ method and normalized to β-actin and control. Briefly, the mean Ct value of the target genes in each sample was normalized to the averaged β-actin Ct values to give a ΔCt value. This was then normalized to control samples (ΔΔCt) and the 2^–ΔΔCt^ value was obtained.

**Table 1:** Quantitative real-time PCR primers

Gene	Primer	Sequence (5′–3′)
*ACAN*	Forward	ACCAGACTGTCAGATACCCC
	Reverse	CATAAAAGACCTCACCCTCC
*SOX-9*	Forward	GCCTCTACTCCACCTTCACC
	Reverse	GTAGACGGGTTGTTCCCAGT
*COL2A1*	Forward	ATTGCCTATCTGGACGAAGC
	Reverse	GCAGTGTACGTGAACCTGCT
*CA12*	Forward	CGTGCTCCTGCTGGTGATCT
	Reverse	AGTCCACTTGGAACCGTTCACT
*KRT19*	Forward	AAGACACACTGGCAGAAACG
	Reverse	GATTCTGCCGCTCACTATCA
*HIF-1α*	Forward	GCCAGACGATCATGCAGCTA
	Reverse	ATCCATTGATTGCCCCAGCA
*MMP-1*	Forward	GGAACAGATACGAAGAGGAAACA
	Reverse	TGTGGGAATCAGAGGTAGAAGA
*MMP-3*	Forward	GCATTGGCTGAGTGAAAGAGAC
	Reverse	ATGATGAACGATGGACAGATGA
*TIMP-1*	Forward	GGTTCCCTGGCATAATCTGAG
	Reverse	ATCGCTCTGGTAGCCCTTCT
*β-ACTIN*	Forward	GTGGGGCGCCCCAGGCACCA
	Reverse	CTTCCTTAATGTCACGCACGATTTC

*ACAN* aggrecan, *COL2A1* collagen II α1, *SOX-9* SRY-related high mobility group-box gene 9, *KRT19* cytokeratin-19, *CA12* carbonic anhydrase XII, *HIF-1α* hypoxia-inducible factor 1α, *MMP* matrix metalloproteinase, *TIMP-1* tissue inhibitor of metalloproteinases-1

### Statistical analysis

SPSS 13.0 software (SPSS Inc., Chicago, IL, USA) was used for statistical analysis. Data are expressed as mean ± standard deviation. Comparisons between two groups were performed by Student’s *t* test. In the experiments with more than two groups, one-way ANOVA followed by the Bonferroni post-hoc test was used. *P* < 0.05 was considered significant.

## Results

### Identification of exosomes derived from BM-MSCs and NPCs

**Fig. 1:**
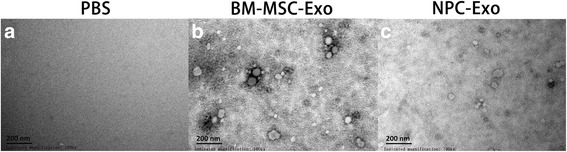
Characterization of BM-MSC and NPC-derived exosomes by TEM. **a** PBS used as a control group; **b** exosomes derived from BM-MSCs; **c** exosomes derived from NPCs. Exosomes were stained with phosphotungstic acid and observed by TEM (×100,000). The two kinds of exosomes were roughly identical, ranging from 30 to 100 nm. *Scale bar* = 200 nm. *BM-MSC* bone marrow mesenchymal stem cell, *Exo* exosomes, *NPC* nucleus pulposus cell, *PBS* phosphate-buffered saline

**Fig. 2:**
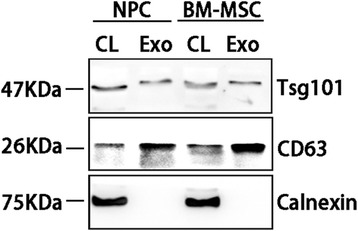
Western blot analyses to test the proteins in exosomes. Western blot analysis was performed on the vesicles to validate exosomal markers CD63, TSG101 and negative protein Calnexin. *NPC* nucleus pulposus cell, *BM-MSC* bone marrow mesenchymal stem cell, *CL* cell lysate, *Exo* exosomes

Exosomes derived from BM-MSCs and NPCs were isolated with the methods described previously [17] and observed by transmission electron microscopy (TEM). The TEM results showed that both cells secreted exosomes and the size of them was roughly identical, with a typical size of 30–100 nm (Fig. 1). Additionally, exosomes from both cells expressed exosomal marker proteins CD63 and TSG101. Calnexin was tested as a negative protein, which is specifically expressed and located in endoplasmic reticulum (Fig. 2).

### Reciprocal uptake of exosomes by BM-MSCs and NPCs

**Fig. 3:**
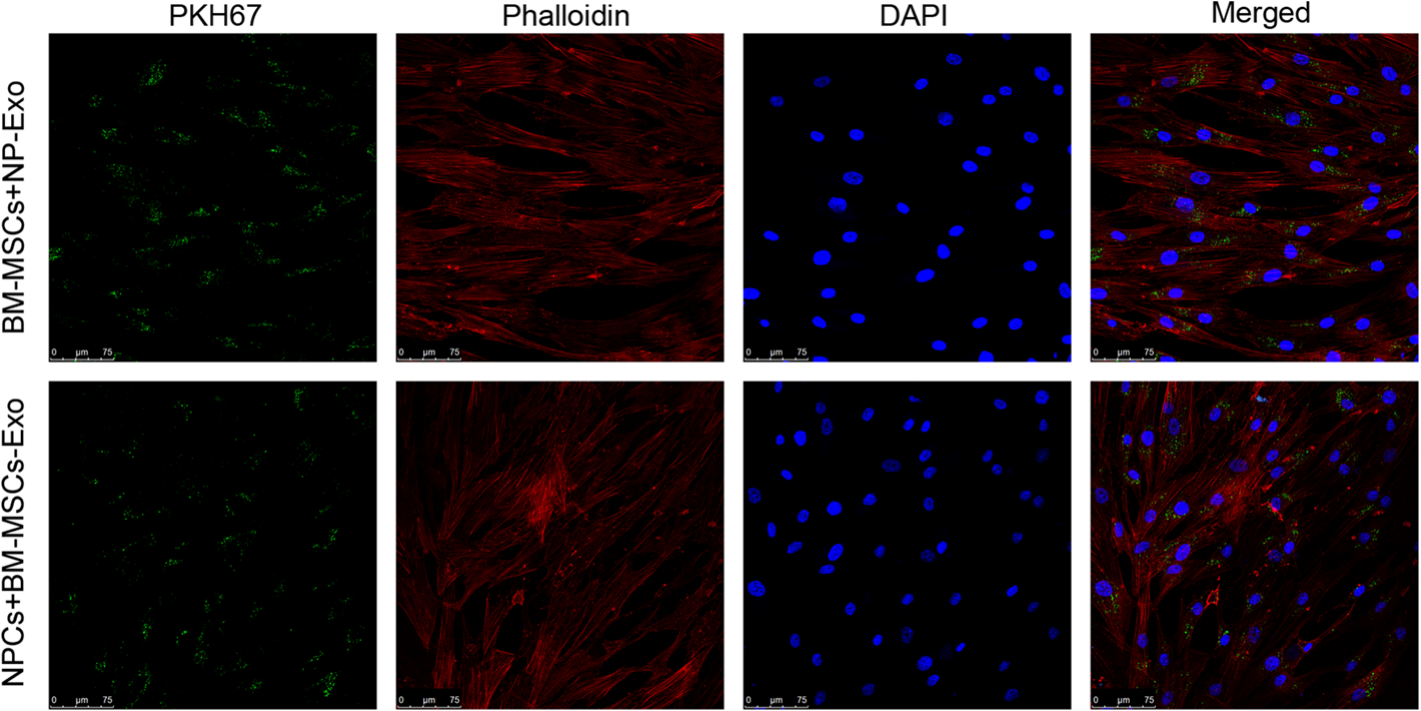
Reciprocal uptake of exosomes by BM-MSCs and NPCs. BM-MSCs and NPCs were respectively incubated with PKH67-labeled exosomes for 4 hours and were observed with a fluorescence confocal microscope. *Green*, PKH67 fluorescence; *red*, phalloidine *blue*; DAPI-stained nuclei. Exosomes were absorbed by responding cells. **BM-MSC** bone marrow mesenchymal stem cell, *Exo* exosomes, *NPC* nucleus pulposus cell

To assay the internalization of exosomes derived from different cells, the PKH67-labeled exosomes were added into the recipient cells. We used a fluorescence confocal microscope to confirm the ability of BM-MSCs to ingest the NPC exosomes and that of NPCs to ingest the BM-MSC exosomes (Fig. 3). After 4-hour incubation of PKH67-labeled exosomes with recipient cells, we observed a punctated green fluorescence in the cells. These results indicate that BM-MSC-derived exosomes can be transferred into NPCs, and equally NPC-derived exosomes can be transferred into BM-MSCs, suggestive of a potential role in the interaction between these cells via exosomes.

### NPC exosomes can induce BM-MSC migration

**Fig. 4:**
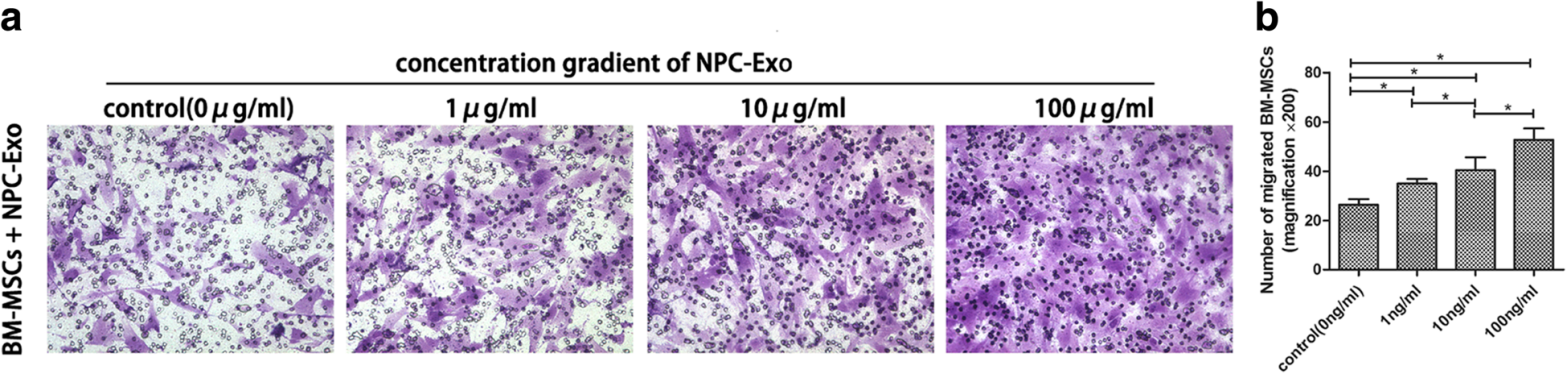
NPC exosome chemotactic assay to BM-MSCs. **a** Migration activity of BM-MSCs treated by NPC exosomes at concentrations of 0, 1, 10 and 100 μg/ml. **b** Number of transmitted cells in the transwell migration assay in (**a**). Values are the mean ± SD. *n* =9. *P < 0.05. *BM-MSC* bone marrow mesenchymal stem cell, *Exo* exosomes, *NPC* nucleus pulposus cell

We further tested whether NPC exosomes could regulate BM-MSC migration, because MSCs need to migrate to IVD in the endogenous repair of degenerate disc. The transwell migration assay revealed that BM-MSCs spontaneously migrated across the transwell membrane. Compared with the control group, the number of migrated cells in the NPC exosome group was significantly increased with the concentration of NPC exosomes (*P* < 0.05) (Fig. 4a, b).

### NPC exosomes induce BM-MSC differentiation toward NP-like cell lineage

**Fig. 5:**
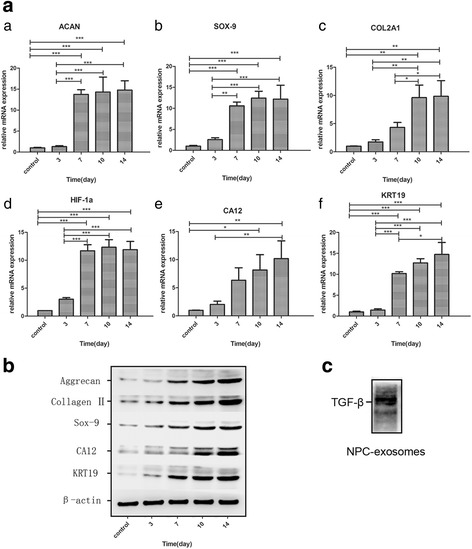
Effects of NPC exosomes on NPC-specific protein expression in BM-MSCs. (**a**) Gene expression of *ACAN* (*a*), *SOX-9* (*b*), *COL2A1* (*c*), *HIF-1α* (*d*), *CA12* (*e*) and *KRT19 (f*) in BM-MSCs was significantly upregulated by NPC exosomes with time. Expression of these genes was normalized to β-actin and control (BM-MSCs alone). Values are the mean ± SD. *n* = 3.*P < 0.05, **P < 0.01, ***P < 0.001. (**b**) NPC-specific protein expression in BM-MSCs described in (**a**) analyzed by western blot analysis. (**c**) TGF-β in NPC exosomes. *NPC* nucleus pulposus cell

Quantitative PCR was used to analyze the mRNA levels of the NP marker genes (*ACAN*, *SOX-9*, *COL2A1*, *HIF-1α*, *CA12*, *KRT19*) in BM-MSCs after incubation with NPC exosomes (final concentration = 50 μg/ml) for 3, 7, 10 and 14 days. Western blot analysis was also performed to determine aggrecan, collagen II, sox-9, hif-1α and ca12 protein expression in BM-MSCs stimulated by NPC exosomes for 3, 7, 10 and 14 days. The qRT-PCR results showed that BM-MSCs on incubation with NPC exosomes showed a significant increase in the mRNA levels of *ACAN*, *SOX-9*, *COL2A1*, *HIF-1α*, *CA12* and *KRT19* compared with BM-MSCs cultured alone (Fig. 5a). Western blot analysis confirmed an increase in protein levels of those genes (Fig. 5b).

### NPC exosomes are more effective to induce BM-MSC differentiation than indirect cocultured system

**Fig. 6:**
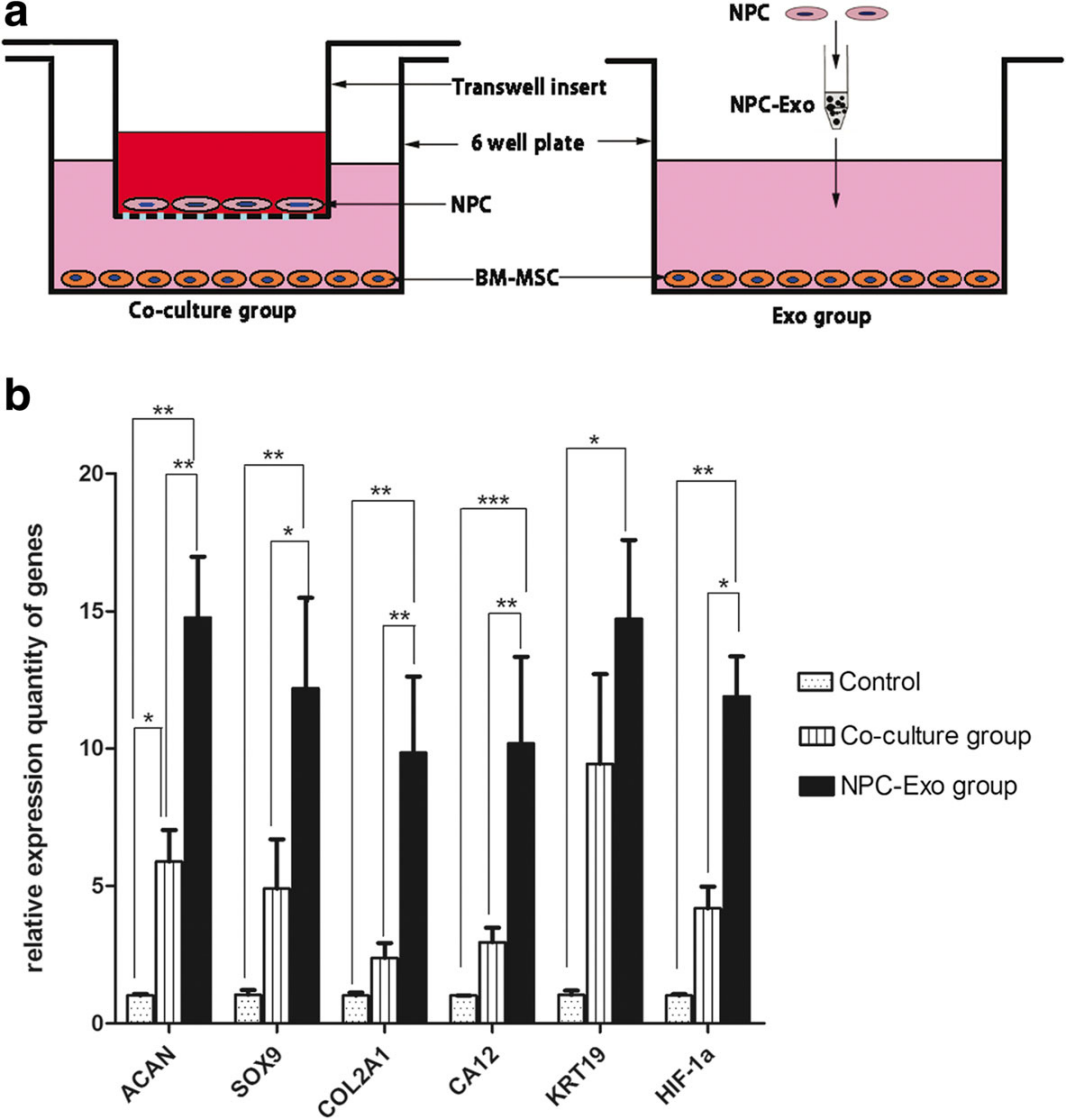
Comparison of gene expression in BM-MSCs after coculture with NPCs and after stimulation by NPC exosomes. **a** Schematic diagram of experimental groups. NPCs and BM-MSCs were cocultured by an indirect method using a transwell system. NPCs were cultured in the upper chamber and BM-MSCs in the lower chamber to coculture for 14 days. NPC exosomes alone were added to BM-MSCs in the Exo group. BM-MSCs without treatment were taken as the control group. **b** After 14 days of treatment, qRT-PCR detected relative mRNA expression in BM-MSCs. Expression levels of *ACAN* (*P* < 0.01), *SOX-9* (*P* < 0.05), *COL2A1* (*P* < 0.01), *HIF-1α* (*P* < 0.01) and *CA12* (*P* < 0.05), but not *KRT19* (*P* > 0.05), mRNA were significantly higher in the Exo group than the coculture group. *P < 0.05, **P < 0.01, ***P < 0.001. *BM-MSC* bone marrow mesenchymal stem cell, *Exo* exosomes, *NPC* nucleus pulposus cell

In the coculture group, a transwell system was used for indirect coculture of NPCs and BM-MSCs. The NPCs (2 × 10^5^/well) were cultured in the upper chamber and the BM-MSCs (2 × 10^5^/well) were in the lower chamber to coculture for 14 days. In the Exo group, the NPC exosomes (final concentration = 50 μg/ml) were added to the BM-MSCs directly. The control group included BM-MSCs alone. We simultaneously detected the gene profile expression of BM-MSCs which were indirectly cocultured with NPCs and directly stimulated by NPC exosomes, shown as a schematic diagram (Fig. 6a, b). Expression of *ACAN*, *SOX-9*, *COL2A1*, *HIF-1α*, *CA12* and *KRT19* was significantly upregulated in the Exo group compared with the coculture group and control. Meanwhile, the levels of *ACAN*, *KRT19* and *HIF-1α* were significantly increased in the coculture group compared with the control group.

The results suggest that NPC exosomes have the ability to induce BM-MSC migration and differentiation into NPCs and that NPC-derived exosomes play an obvious action in the coculture system in BM-MSC differentiation along the NPC lineage. Previous studies have shown that TGF-β is one of the factors that can induce BM-MSCs differentiating toward NPC-like cells. We found the TGF-β is highly expressed in NPC exosomes (Fig. 5c).

Because of the beneficial effect of BM-MSCs to degenerate NPCs determined previously, the therapeutic action of BM-MSC exosomes to degenerate NPCs was evaluated by two indicators: the NPC proliferation rate and extracellular matrix gene expression after treatment by BM-MSC exosomes.

### BM-MSC exosomes promote NPC proliferation

**Fig. 7:**
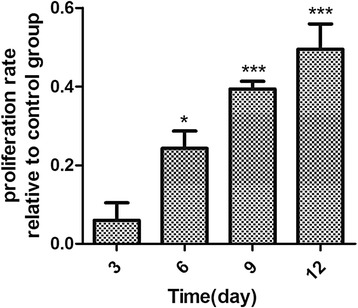
NPC proliferation rate increased with the time for which NPCs were stimulated by BM-MSC exosomes. The group of day 12 has the greatest relative proliferation rate (49% compared with control). The day 6, day 9 and day 12 groups had significant difference from the control group (*P < 0.05, ***P < 0.001). The day 3 group had no significant statistical difference (*P* > 0.05). Results shown as the relative proliferation rate to the control group

The CCK-8 assay showed that, after treatment of BM-MSC exosomes, the NPC proliferation rate increased with duration of interaction. The groups of day 6 (*P* < 0.01), day 9 (*P* < 0.001) and day 12 (*P* < 0.001) had significant difference from the control group; the day 12 group had the highest proliferation rate, while the day 3 group had no significant difference from the control group (*P* > 0.05; Fig. 7).

### BM-MSC exosomes improve gene expression in degenerate NPCs

The matrix composition of the IVD is of significance to its function. If the matrix degrades, this can result in reduced disc height (due to loss of matrix) and blood vessel and nerve ingrowth into the normally avascular and aneural IVD [20]. The major degradation process is mediated by matrix metalloproteinases (MMPs). MMPs are normally secreted in a latent form, requiring activation for proteolytic activity and are inhibited by specific tissue inhibitors (TIMPs) [21]. MMPs and TIMPs keep balance in healthy IVD to maintain normal metabolism.

**Fig. 8:**
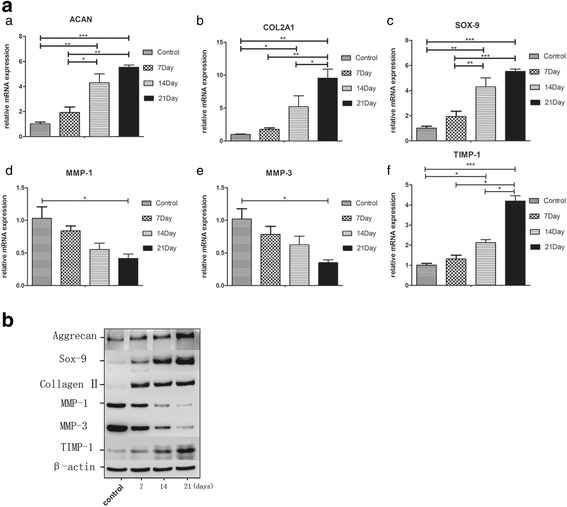
Extracellular matrix expression of degenerate NPCs after NPCs were stimulated by BM-MSC exosomes. **a** qRT-PCR analysis showed an increase in ACAN (*a*), COL2A1 (*b*), SOX-9 (*c*) and TIMP-1 (*f*) and a decrease in MMP-1 (*d*) and MMP-3 (*e*) mRNA expression with the time of stimulation by BM-MSC exosomes. *P < 0.05, **P < 0.01, ***P < 0.001. **b** Western blot analysis showed that the effect of BM-MSC exosomes on NPCs in the protein profile was almost the same as gene expression

To evaluate whether the degenerate NPC gene can be modulated toward a healthy NP gene phenotype, we used quantitative PCR and western blot analysis to determine the expression of aggrecan, collagen II and sox-9, mediators of matrix degradation MMP-1 and MMP-3, and matrix protection molecule TIMP-1 (Fig. 8a, b). The results showed that expression levels of anabolic/matrix protective genes (aggrecan, collagen II, sox-9) were upregulated after NPCs were stimulated by BM-MSC exosomes, and the levels of matrix degrading genes such as MMP-1 and MMP-3 decreased.

## Discussion

One important feature of IVD degeneration is a decrease in viable and functional cell numbers, with a substantial proportion of cells existing in a senescent state. Stem cell-based therapies for repairing the degenerate IVD aim at increasing the number of viable cells capable of producing an appropriate and functional NP-like matrix. The main type of stem cells in application for degenerate NP restoration is MSCs, which have the capacity to differentiate into NP-like cells in vitro. Previous studies have suggested that in direct cell–cell contact, indirect contact or noncontact coculture systems, MSCs could differentiate along the NP-like phenotype and induce the proliferation of NPCs [5, 14, 22–25]. Yuan et al. [26] showed that the NP cell-derived extracellular matrix also has the ability to induce the differentiation of mesenchymal stem cells. Furthermore, Strassburg et al. [27] report that the cell fusion and formation of gap junctions are not the predominant mechanisms of interaction, while transfer of membrane components is operational during direct coculture of MSCs and NP cells. However, the mechanisms by which NPCs and MSCs interact in these systems are currently unclear.

During the past few years, it has been widely accepted that exosomes are important vesicles in intercellular communication for modulating or mediating cellular processes and the function of exosomes is highly correlative with their origin and the loaded component. Therefore, we speculated that the NPC exosomes containing NPC information target MSCs and then influence MSC activity. Our study showed that NPC exosomes could induce BM-MSCs to differentiate toward NP-like cells, and the effectiveness of exosomes was stronger compared with an indirect noncontact coculture system. Meanwhile, previous studies have shown that the recruitment of cells from the surrounding environment to replenish the viable and functional cells is an important aspect of the regenerative process of IVD. The progenitor cells and stem cell niches inside and around IVDs, as well as those in the blood and bone marrow system, play pivotal roles in maintaining physiological homeostasis and the endogenous repair of IVDs during tissue repair [28]. Previous studies showed that microparticles can activate MSC migration and cytokine secretion. These results have potential applications toward elucidating the mechanisms underlying MSC homing to and modulation of injury sites [29]. To examine whether the NPC exosomes can recruit BM-MSCs from the surrounding environment to repair the degenerative NPCs, we tested the NPC exosome chemotaxis to BM-MSCs and the result was positive. These results indicate that in the process of IVD degeneration, NPC exosomes can recruit BM-MSCs and the recruited BM-MSCs can different along the NP lineage, which may replenish the IVD cells and produce appropriate extracellular matrix. Besides, there may be other interactions between NPCs and other cells inside the intervertebral disc such as annulus fibrosus [30] or cartilage end plate cells. Further studies should be done to reveal how the exosomes work in the process of IVD degeneration.

On the other hand, MSCs, whose mechanism of action is predominantly paracrine, are being widely used for the treatment of a variety of human diseases. However, no factor has been proven sufficient to mediate the therapeutic effects of MSCs [10]. Exosomes are appealing candidates as vectors of MSC efficacy. MSC exosomes have the versatility and capacity to interact with multiple cell types within the immediate vicinity and remote areas, so as to elicit appropriate cellular responses. Several studies have reported that MSC exosomes have functions similar to those of MSCs, such as repairing tissue damage, suppressing inflammatory responses and modulating the immune system [31, 32]. In our study, we investigated the proliferation of NPCs and extracellular gene expression in degenerate NPCs after being stimulated by BM-MSC exosomes. The degenerate NPC proliferation was obviously promoted compared with the control, and the gene expression of aggrecan, collagen II and sox-9 also increased significantly. These results suggest that degenerate NPCs induced by BM-MSC exosomes can improve the proliferation activity and generate a healthier extracellular matrix. Meanwhile, the downregulation of MMP-1/MMP-3 and upregulation of TIMP-1 indicates a more balanced anabolism/catabolism. According to the results, we can propose that the exosomes from MSCs have potential to act as a new therapeutic method in DDD.

In addition, the exosomes derived from MSCs may be a more appropriate candidate than MSCs in stem cell-based therapies. The central portion of degenerating discs contains low cellularity, low glucose, high osmolarity, low pH, high mechanical variations and low oxygen in addition to an inflammatory environment. Despite the effective influences of MSCs to degenerate IVD, even cell-injection strategies showing positive effects [33, 34], there remain obstacles to MSC application in the clinic, especially the problem of how the transplanted cells are able to survive and adapt in the avascular IVD [25, 35]. Compared with MSCs, exosomes are more stable and reservable, and may survive a hostile environment, providing an alternative therapy for various diseases.

Exosomes are extracellular vesicles which consist of proteins, lipids and RNAs in various forms. We investigated the exosomes as a holistic object, so specific components were not investigated in this study. Further research is expected on the specific component of NPC and MSC exosomes including proteins, microRNA, transcription factors and lipids [36]. In addition, our experiments were conducted in vitro; further study is expected to verify the feasibility of MSC exosome therapy for DDD or even other diseases in vivo.

## Conclusion

In the present study, we investigated the role of exosomes derived from BM-MSCs and NPCs in cellular interactions. NPC exosomes can stimulate BM-MSCs to differentiate to an NP-like phenotype, and BM-MSC exosomes also stimulate the degenerate NPC population to regain a nondegenerate phenotype and consequently enhance matrix synthesis for self-repair. Our study suggests that exosomes may play key roles in IVD endogenous repair and current stem cell therapies for intervertebral disc degeneration. Moreover, given a variety of functions and multiple advantages, exosomes—alone or as vehicles of genes and drugs—have the potential to be used in cell-free therapy.

## Abbreviations

ACAN: Aggrecan
AF: Annular fibers
BM-MSC: Bone marrow mesenchymal stem cell
CA12: Carbonic anhydrase XII
CCK-8: Cell Counting Kit-8
CEP: Cartilaginous endplate
COL2A1: Collagen II α1
DDD: Degenerative disc disease
DMEM: Dulbecco’s modified Eagle medium
Exo: Exosomes
GBD: Global Burden of Disease
HIF-1α: Hypoxia-inducible factor 1α
HSP: Heat shock protein
ILV: Intraluminal vesicle
KRT19: Cytokeratin-19
LAS AF: Leica Application Suite Advanced Fluorescence
LBP: Lower back pain
MMP: Matrix metalloproteinase
MSC: Mesenchymal stem cell
MVB: Exocytosis of multivesicular body
NPC: Nucleus pulposus cell
OD: Optical density
PBS: Phosphate-buffered saline
SOX-9: SRY-related high mobility group-box gene 9
TEM: Transmission electron microscope
TIMP-1: Tissue inhibitors of metalloproteinases-1
YLDs: Years lived with disability
